# Impact of Four-Phonon Scattering on Thermal Transport and Thermoelectric Performance of Penta-XP_2_ (X = Pd, Pt) Monolayers

**DOI:** 10.3390/nano15181396

**Published:** 2025-09-11

**Authors:** Yangshun Lan, Yueyu Zhang, Honggang Zhang, Ping Wang, Ning Wang, Yangjun Yan, Xiaoting Zha, Changchun Ding, Yuzhi Li, Chuanfu Li, Yunjun Gu, Qifeng Chen

**Affiliations:** 1 Key Laboratory of High Performance Scientific Computation, School of Science, Xihua University, Chengdu 610039, China; 2 School of Physics and Electronic Engineering, Sichuan University of Science & Engineering, Yibin 644000, China; 3 National Key Laboratory for Shock Wave and Detonation Physics Research, Institute of Fluid Physics, Chinese Academy of Engineering Physics, Mianyang 621900, China; 4 School of Mathematics and Physics, Southwest University of Science and Technology, Mianyang 621010, China

**Keywords:** thermal transport, thermoelectric, penta-2D materials, DFT, four-phonon scattering

## Abstract

Accurately understanding and modulating thermal and thermoelectric transport in penta-XP_2_ (X = Pd, Pt) monolayers is crucial for their applications in nanoelectronics and energy conversion. We systematically investigate the thermal conductivity and thermoelectric properties of penta-XP_2_ monolayers through first-principles calculations, incorporating four-phonon (4ph) scattering and electron–phonon interaction (EPI) effects. The 4ph scattering, particularly Umklapp and redistribution processes, markedly suppresses lattice thermal conductivity by generating substantial thermal resistance and disrupting phonon population distributions. At 300 K, the lattice thermal conductivity is reduced to 0.87 W/mK (80% reduction) for penta-PdP_2_ and 1.64 W/mK (79% reduction) for penta-PtP_2_ compared to three-phonon-only scattering. Combining this with EPI-optimized electronic transport yields enhanced thermoelectric figures of merit (*ZT*), increasing from 0.21 to 0.86 for penta-PdP_2_ and from 0.11 to 0.34 for penta-PtP_2_, alongside a broadened optimal carrier concentration range. These findings highlight momentum-conserving 4ph scattering as a key mechanism for phonon transport modulation and thermoelectric efficiency improvement in penta-XP_2_ materials, providing theoretical guidance for designing high-performance nanoscale thermal management and energy conversion devices.

## 1. Introduction

The global energy crisis has become one of the most critical challenges of the 21st century, prompting extensive efforts to develop sustainable energy alternatives and high-efficiency energy-saving materials [1,2,3,4,5,6]. Thermoelectric materials have attracted considerable interest among diverse energy technologies because they can directly and reversibly transform waste heat into electrical power [7]. With advantages such as all-solid-state operation, noiseless performance, high reliability, and miniaturization capability, thermoelectric materials demonstrate strong potential in applications like electronic cooling [7], industrial waste heat recovery [8], aerospace power systems [9], and wearable devices [10]. The thermoelectric efficiency is typically evaluated by the dimensionless figure of merit $ZT=S^{2}\sigma T/\kappa$, with higher *ZT* values achieved through enhanced power factors ($PF=S^{2}\sigma$) and suppressed thermal conductivity (*κ*). Optimizing these transport properties simultaneously poses significant challenges due to their intrinsic interdependence. To overcome this, strategies such as carrier concentration tuning [11], band structure engineering [12], nanostructuring [13], interface [14], and alloying [15] have been proposed to decouple the interdependent transport parameters. Layered semiconductors are regarded as attractive thermoelectric materials owing to their quantum confinement effects and enhanced phonon scattering [16], which help to decouple electrical and thermal transport and thereby improve thermoelectric performance.

Among various low-dimensional materials, layered pentagonal two-dimensional materials (penta-2DMs) have drawn broad attention due to their unique lattice topology, moderate band gaps, high air stability, and intrinsic anisotropy [17,18,19]. These characteristics give rise to excellent mechanical, electronic, and optical properties, rendering them attractive for thermoelectric applications [17]. Most recently, several noble-metal-based penta-materials, such as Pt and Pd, have demonstrated remarkable thermoelectric performance. Penta-PdX_2_ and penta-PtX_2_ (X = S, Se, Te) have been reported to exhibit high Seebeck coefficients, low lattice thermal conductivity, and favorable power factors, with room-temperature *ZT* values surpassing that of commercial Bi_2_Te_3_ [20,21,22]. Among them, the penta-monolayers PdP_2_ and PtP_2_, representative members of the Pd/Pt-based XP_2_ (X = Pd, Pt) monolayers, have emerged as promising candidates for thermoelectric applications. The penta-XP_2_ (X = Pd, Pt) monolayers exhibit dynamic stability, direct band gaps around 0.3–0.8 eV, ultrahigh carrier mobilities (~10^5^ cm^2^·V^−1^·s^−1^), and intrinsically low lattice thermal conductivities [23,24,25,26,27]. Their room-temperature *ZT* values have been reported in the range of 0.06 to 0.34, depending on the doping level and chalcogen species, with p-type Pd-based monolayers exhibiting particularly favorable performance [28,29,30]. These thermoelectric properties are closely associated with strong anharmonic lattice dynamics, as evidenced by flat phonon branches and large acoustic–optical phonon gaps. Such features suggest that conventional three-phonon (3ph) scattering mechanisms may be inadequate to fully describe phonon–phonon interactions, and four-phonon (4ph) scattering processes could play a significant or even dominant role [31,32]. The enhancement of such higher-order anharmonic interactions can substantially affect thermal transport and limit further improvement in thermoelectric efficiency [33,34]. For the electronic transport, relying solely on the deformation potential theory is inadequate for accurately estimating *ZT*, as it omits detailed electron–phonon interaction (EPI) effects, which can critically influence carrier mobility and electrical conductivity [35,36]. Therefore, a comprehensive understanding and accurate modeling of both 4ph and EPI effects are essential for optimizing the thermoelectric performance of penta-XP_2_ (X = Pd, Pt) and promoting their application in energy conversion and next-generation electronic devices.

With this in mind, we conduct comprehensive first-principles calculations incorporating 4ph scattering and EPI effects to investigate the thermal transport and thermoelectric properties of penta-XP_2_ (X = Pd, Pt) monolayers. By analyzing phonon dispersion, group velocity, and thermal conductivity under 3ph and combined 3ph + 4ph scattering, together with electronic transport coefficients, the intrinsic thermoelectric transport behavior is systematically elucidated. The results show that 4ph scattering contributes on a scale comparable to 3ph processes, with Umklapp and redistribution processes acting as the dominant phonon-limiting mechanisms. These processes markedly suppress lattice thermal conductivity and shorten phonon mean free paths, which, in turn, enhance the thermoelectric figure of merit and broaden the optimal carrier concentration range. Our present study offers microscopic insight into 4ph phonon scattering in penta-XP_2_ monolayers and provides a theoretical basis for optimizing thermal management and thermoelectric performance in nanoscale energy and electronic devices.

## 2. Computational Methods

The first-principles calculations were performed using density functional theory (DFT) within the generalized gradient approximation (GGA) of Perdew–Burke–Ernzerhof (PBE) [37], as implemented in the Vienna Ab initio Simulation Package (VASP) [38,39]. The Brillouin zone was sampled using a 15 × 15 × 1 Monkhorst–Pack mesh [40], and a plane-wave cutoff energy of 600 eV was applied [41]. A vacuum spacing of 20 Å was introduced along the z-direction to avoid interactions between periodic images. The electronic and ionic relaxations convergence criteria were set to 10^−7^ eV and 10^−4^ eV Å^−1^. The thermal stability of penta-XP_2_ (X = Pd, Pt) was assessed via ab initio molecular dynamics (AIMD) simulations in the canonical ensemble (NVT) [42], using a 4 × 4 × 1 supercell for 20,000 steps with a time step of 2 fs. The lattice thermal conductivity *κ*_l_ was evaluated by iteratively solving the phonon Boltzmann transport equation (BTE) in the relaxation time approximation, implemented via the ShengBTE [43] and FourPhonon [44] codes. The harmonic phonon dispersions and second-order interatomic force constants (IFCs) are obtained from density functional perturbation theory (DFPT) [45] within a 5 × 5 × 1 supercell. For third- and fourth-order anharmonic IFCs, 4 × 4 × 1 and 3 × 3 × 1 supercells were adopted, with the interaction range extending up to the fifth-nearest neighbors for the third-order IFCs and the third-nearest neighbors for the fourth-order IFCs, respectively. The *κ_l_* tensor is computed based on the phonon BTE [46,47,48] as

(1) $$\kappa_{l}^{\alpha \beta }=\frac{1}{k_{b}T^{2}VN}\sum_{\lambda }n_{0}\left(n_{0}+1\right){\left(\hbar \omega_{\lambda }\right)}^{2}v_{\lambda }^{\alpha }F_{\lambda }^{\beta },$$

where the *k_b_*, *V*, *N*, and *ℏ* denote the Boltzmann constant, the system volume, the number of wave vectors, and the reduced Planck constant, respectively. The index *λ* refers to a specific phonon mode, which includes both the phonon branch and wave vector, and the indices *α* and *β* represent Cartesian directions. *ω_λ_* is the phonon frequency, and $v_{\lambda }^{\alpha }$ denotes the phonon group velocity along the *α*-direction. The $F_{\lambda }^{\beta }$ is written as

(2) $$F_{\lambda }^{\beta }=\tau_{\lambda }^{0}\left(\nu_{\lambda }^{\beta }+\Delta_{\lambda }\right),$$

where $\tau_{\lambda }^{0}$ and $\Delta_{\lambda }$ stand for the phonon lifetime and the deviation function under the single-mode relaxation time approximation (SMRTA). The 4ph scattering rates (SRs) are expressed as

(3) $$\begin{array}{l} \Gamma_{\lambda \lambda^{\prime }\lambda^{\prime \prime }\lambda^{\prime \prime \prime }}^{\left(++\right)}=\frac{\hbar^{2}\pi }{8N}\frac{\left(1+n_{\lambda^{\prime }}^{0}\right)\left(1+n_{\lambda^{\prime \prime }}^{0}\right)n_{\lambda^{\prime \prime \prime }}^{0}}{n_{\lambda }^{0}}{\left|V_{\lambda \lambda^{\prime }\lambda^{\prime \prime }\lambda^{\prime \prime \prime }}^{\left(++\right)}\right|}^{2}\frac{\delta \left(\omega_{\lambda }+\omega_{\lambda^{\prime }}+\omega_{\lambda^{\prime \prime }}-\omega_{\lambda^{\prime \prime \prime }}\right)}{\omega_{\lambda }\omega_{\lambda^{\prime }}\omega_{\lambda^{\prime \prime }}\omega_{\lambda^{\prime \prime \prime }}}nn\\ \Gamma_{\lambda \lambda^{\prime }\lambda^{\prime \prime }\lambda^{\prime \prime \prime }}^{\left(+-\right)}=\frac{\hbar^{2}\pi }{8N}\frac{\left(1+n_{\lambda^{\prime }}^{0}\right)n_{\lambda^{\prime \prime }}^{0}n_{\lambda^{\prime \prime \prime }}^{0}}{n_{\lambda }^{0}}{\left|V_{\lambda \lambda^{\prime }\lambda^{\prime \prime }\lambda^{\prime \prime \prime }}^{\left(+-\right)}\right|}^{2}\frac{\delta \left(\omega_{\lambda }+\omega_{\lambda^{\prime }}-\omega_{\lambda^{\prime \prime }}-\omega_{\lambda^{\prime \prime \prime }}\right)}{\omega_{\lambda }\omega_{\lambda^{\prime }}\omega_{\lambda^{\prime \prime }}\omega_{\lambda^{\prime \prime \prime }}}\\ \Gamma_{\lambda \lambda^{\prime }\lambda^{\prime \prime }\lambda^{\prime \prime \prime }}^{\left(--\right)}=\frac{\hbar^{2}\pi }{8N}\frac{n_{\lambda^{\prime }}^{0}n_{\lambda^{\prime \prime }}^{0}n_{\lambda^{\prime \prime \prime }}^{0}}{n_{\lambda }^{0}}{\left|V_{\lambda \lambda^{\prime }\lambda^{\prime \prime }\lambda^{\prime \prime \prime }}^{\left(--\right)}\right|}^{2}\frac{\delta \left(\omega_{\lambda }-\omega_{\lambda^{\prime }}-\omega_{\lambda^{\prime \prime }}-\omega_{\lambda^{\prime \prime \prime }}\right)}{\omega_{\lambda }\omega_{\lambda^{\prime }}\omega_{\lambda^{\prime \prime }}\omega_{\lambda^{\prime \prime \prime }}} \end{array},$$

where $V_{\lambda \lambda^{\prime }\lambda^{\prime \prime }\lambda^{\prime \prime \prime }}$ is the transition probability matrices. The superscripts of (++), (+−), and (−−) correspond to the combination (λ + λ′ + λ″ → λ‴), redistribution (λ + λ′ → λ″ + λ‴), and splitting (λ → λ′ + λ″ + λ‴) processes in 4ph scattering, respectively.

The electronic transport properties, including electron–phonon interactions (EPI), were evaluated using the PERTURBO code [49], which utilizes electronic and phonon inputs generated from QUANTUM ESPRESSO (QE) calculations [50,51]. These calculations were conducted with a kinetic energy cutoff of 80 Ry and an energy convergence threshold of 10^−10^ Ry. A 16 × 16 × 1 *k*-point mesh was employed for electronic calculations, while an 8 × 8 × 1 *q*-point mesh was applied for phonon dispersion. Maximally localized Wannier functions were constructed by projecting the density of states onto the *s*, *p*, and *d* orbitals of Pd/Pt atoms and the *s* and *p* orbitals of P atoms. To ensure convergence, PERTURBO simulations utilized dense 400 × 400 × 1 *k*-point and 200 × 200 × 1 *q*-point meshes for electronic transport calculations. The relaxation time was evaluated as the inverse of the scattering rate Γ_nk_, i.e.,$\tau_{nk}=\;\Gamma_{nk}^{-1}$, where Γ_nk_ was the scattering rate, given by

(4) $$\Gamma_{nk}=\frac{1}{N_{q}}\sum_{m,vq}W_{nk,mk+q}^{vq},$$

where $W_{nk,mk+q}^{vq}$ is the scattering probability, accounting for both phonon emission and absorption processes, and *N*_q_ is the total number of phonon *q*-points used in the Brillouin zone sampling. The electrical conductivity (*σ*) was calculated from the transport distribution function using the expression

(5) $$\sigma_{\alpha \beta }=e^{2}\int \Sigma_{\alpha \beta }(\varepsilon )\left(-\partial f^{0}/\partial \varepsilon \right)d\varepsilon ,$$

where $\Sigma_{\alpha \beta }(\varepsilon )$ is the transport distribution function (TDF) at energy *ε*, defined as [49]

(6) $$\Sigma_{\alpha \beta }(\varepsilon )=\frac{S}{N_{k}V}\sum_{n,k}v_{nk,\alpha }F_{nk}^{\beta }\delta (\varepsilon -\varepsilon_{nk}),$$

where *S*, *V*, and *N*_k_ are the spin degeneracy, unit cell volume, and number of *k*-points, respectively, and the *v*_nk,α_ is the group velocity component along the *α* direction. The $F_{nk}^{\beta }$ characterizes the first-order deviation from equilibrium of the electron distribution,

(7) $$F_{nk}^{\beta }=\tau_{nk}v_{nk,\beta }+\frac{\tau_{nk}}{N_{q}}\sum_{m,\nu q}W_{nk,mk+q}^{\nu q}F_{m,k+q}^{\beta }.$$

The TDF therefore combines the effects of band dispersion, scattering, and the density of available states, linking the electronic structure with macroscopic transport coefficients. The Seebeck coefficient (*S*) was obtained as [35]

(8) $$S_{\alpha \beta }=\frac{1}{eT}\frac{\int \sum_{\alpha \beta }\left(\varepsilon \right)(\varepsilon -\varepsilon_{F})\left(-\partial f^{0}/\partial \varepsilon \right)d\varepsilon }{\int \sum_{\alpha \beta }\left(\varepsilon \right)\left(-\partial f^{0}/\partial \varepsilon \right)d\varepsilon },$$

where *ε*_F_ represents the Fermi level corresponding to a specific doping concentration, and *T* is the absolute temperature. The charge carrier contribution to electronic thermal conductivity *κ*_e_ was calculated using [52]

(9) $$\kappa_{\alpha \beta }^{e}=\frac{1}{T}\left\{\int \sum_{\alpha \beta }\left(\varepsilon \right){\left(\varepsilon -\varepsilon_{F}\right)}^{2}\left(-\partial f^{0}/\partial \varepsilon \right)d\varepsilon -\frac{{\left(\int \sum_{\alpha \beta }\left(\varepsilon \right)\left(\varepsilon -\varepsilon_{F}\right)\left(-\partial f^{0}/\partial \varepsilon \right)d\varepsilon \right)}^{2}}{\int \sum_{\alpha \beta }\left(\varepsilon \right)\left(-\partial f^{0}/\partial \varepsilon \right)d\varepsilon }\right\}.$$

## 3. Results

**Figure 1:**
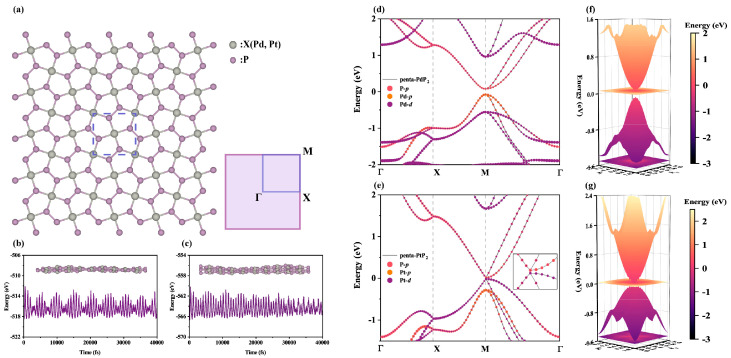
(**a**) Top view illustration of the penta-XP_2_ (X = Pd, Pt) monolayer, highlighting the unit cell using a dashed rectangle. The corresponding 2D Brillouin zone and high-symmetry paths are shown on the right. Total energy fluctuations of penta-PdP_2_ (**b**) and penta-PtP_2_ (**c**) during AIMD simulations at 300 K, and the insets display the side views of the final atomic configurations. Orbital-projected band structures of penta-PdP_2_ (**d**) and penta-PtP_2_ (**e**), highlighting contributions from P-*p*, Pd/Pt-*p*, and Pd/Pt-*d* orbitals. The inset of (**e**) highlights the band features near the Fermi level. Three-dimensional energy surfaces near the Fermi level, illustrating the lowest conduction and highest valence bands for penta-PdP_2_ (**f**) and penta-PtP_2_ (**g**), respectively. The projection plane below the valence and conduction bands illustrates the variation rate of the band structure.

As illustrated in Figure 1a, the optimized structures of penta-XP_2_ (X = Pd, Pt) exhibit a planar pentagonal network composed entirely of five-membered rings, forming a Cairo tiling-like pattern in the top view [53]. The unit cell, outlined by a dashed rectangle, consists of two X atoms and four P atoms and belongs to the P4/mbm space group (No. 127), exhibiting high crystallographic symmetry. Every X atom bonds with four P atoms, giving rise to a characteristic pentagonal configuration. The two monolayers exhibit similar structural features, with optimized lattice constants of 5.86 Å for penta-PdP_2_ and 5.83 Å for penta-PtP_2_, which differ by less than 0.5% from previous literature reports [25,26,27,28,29]. The right panel of Figure 1a shows the corresponding two-dimensional Brillouin zone and high-symmetry path (Γ-X-M-Γ), which are used in subsequent electronic and phononic analyses. To assess thermal stability, ab initio molecular dynamics (AIMD) simulations were carried out at 300 K with 4 × 4 × 1 supercells for a total simulation time of 40 ps. Figure 1b and Figure 1c display the total energy fluctuations of penta-PdP_2_ and penta-PtP_2_ during the simulation, respectively. Both penta-XP2 exhibit relatively small energy fluctuations and maintain structural integrity throughout the simulation, with no significant reconstruction or bond breaking observed. The insets show side views of the final configurations, further confirming their room-temperature stability. Notably, penta-PdP_2_ displays slightly smaller energy fluctuations than penta-PtP_2_, indicating superior thermal robustness under identical conditions.

The orbital-resolved electronic band structures of penta-XP_2_ (X = Pd, Pt) are shown in Figure 1d,e. Both materials exhibit direct bandgaps located at the M point, with values of 0.15 eV for penta-PdP_2_ and 0.03 eV for penta-PtP_2_. The conduction and valence band edges mainly originate from the P-*p* orbitals. For penta-PdP_2_, additional contributions from Pd-*p* states are observed near the valence band maximum, whereas the corresponding states in penta-PtP_2_ are largely governed by Pt-*d* orbitals. This difference arises from the higher energy level and stronger hybridization tendency of the Pt 5*d* orbitals. As shown in Figure S1, the inclusion of spin–orbit coupling (SOC) induces negligible changes in the overall band structure, and the use of the HSE06 hybrid functional primarily increases the bandgap without affecting the locations of the conduction band minimum (CBM) and valence band maximum (VBM), consistent with previous results [29]. Therefore, the influences of SOC and HSE06 can be neglected and will not be incorporated into our subsequent calculations. To support accurate electron–phonon interaction calculations, maximally localized Wannier functions were constructed, as presented in Figure S1a,c. The interpolated bands show excellent agreement with the first-principles results, particularly near the band edges. The three-dimensional band dispersion of the lowest conduction and highest valence bands, shown in Figure 1f,g, reveals smooth and nearly circular isoenergy contours near the band edges. This indicates that penta-XP_2_ (X = Pd, Pt) exhibits in-plane isotropic electronic transport behavior, which is a favorable characteristic for thermoelectric and low-power electronic applications.

**Figure 2:**
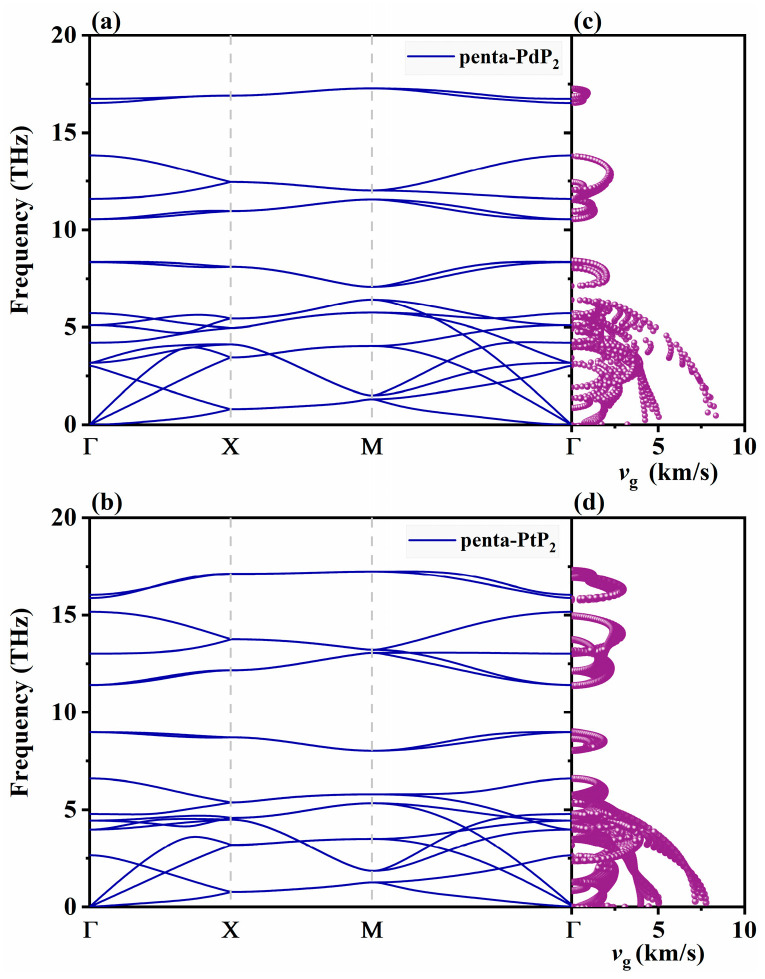
The phonon dispersions (**a**,**b**), and mode-resolved phonon group velocity (**c**,**d**) of penta-PdN_2_ and penta-PtN_2_ monolayers.

Thermal conductivity serves as an essential parameter in evaluating the thermoelectric efficiency of materials. In semiconductors, thermal transport is primarily dominated by phonon–phonon interactions, making accurate prediction of lattice thermal conductivity (*κ*_l_) essential for evaluating their practical applications. To assess the dynamical stability of monolayer penta-XP_2_ (X = Pd, Pt), phonon dispersion relations are plotted in Figure 2a,b. The dynamic stability of both structures is validated by the fact that no imaginary modes appear within the Brillouin zone. The slope of the phonon dispersion provides important information about the phonon group velocity (*v*_g_), which is a key factor influencing thermal transport. As depicted in Figure 2c,d, both materials exhibit relatively high *v*_g_ in the acoustic branches, particularly in the low-frequency region, which is favorable for thermal transport. The comparable magnitude of *v*_g_ between penta-PdP_2_ and penta-PtP_2_ suggests similar phonon propagation efficiency in both materials.

**Figure 3:**
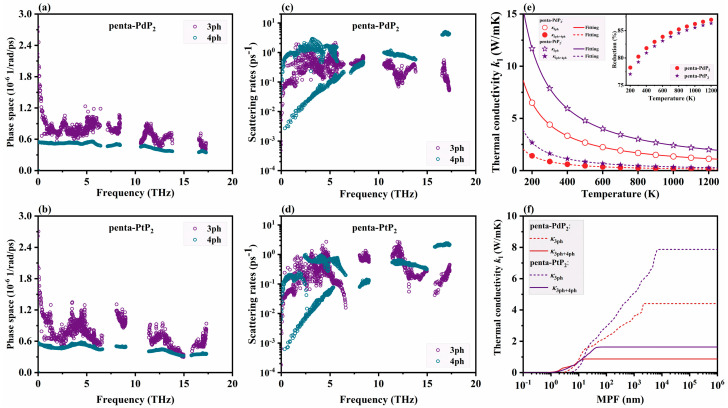
Frequency-resolved phase space (**a**,**b**) and phonon scattering rates (**c**,**d**) of monolayer penta-XP_2_ (X = Pd, Pt) at 300 K, considering both three-phonon (3ph) and four-phonon (4ph) processes. (**e**) Temperature-dependent lattice thermal conductivity *κ*_l_ of penta-XP_2_ (X = Pd, Pt) monolayer calculated under three-phonon *κ*_3ph_ and combined three- and four-phonon *κ*_3ph+4ph_ scattering processes. The symbols represent the results obtained by solving the BTE, and the solid and dashed lines denote the corresponding temperature-dependent fitting curves. The inset shows the percentage reduction of *κ*_l_ when 4ph scattering is included. (**f**) Cumulative thermal conductivity versus phonon mean free path (MFP) under different scattering mechanisms at 300 K.

According to the phonon Boltzmann transport equation (BTE), in addition to the phonon group velocity (*v*_g_), the lattice thermal conductivity (*κ*_l_) is also influenced by the available phase space and phonon scattering rates. The phonon scattering phase space reflects the statistical likelihood of allowed scattering processes that obey both energy and momentum conservation. As shown in Figure 3a,b, the phase space for 4ph interactions (*P*_4_) in penta-XP_2_ (X = Pd, Pt) is generally smaller than that of the 3ph processes (*P*_3_) across the frequency spectrum, indicating the dominance of three-phonon processes in terms of accessible scattering channels. Nevertheless, the non-negligible magnitude of *P*_4_ in both penta-XP_2_ highlights that 4ph processes play an active role in phonon scattering and should be considered for accurate thermal transport analysis. The importance of 4ph interactions is further revealed by the mode-resolved scattering rates shown in Figure 3c,d. Penta-XP_2_ (X = Pd, Pt) exhibit comparable 3ph and 4ph scattering rates across most of the frequency range, and the 4ph scattering rates of penta-PdP_2_ even exceed the 3ph ones in the low-frequency region. Although the *P*_4_ of penta-XP_2_ is generally smaller than that of *P*_3_, the comparable scattering strength across a broad frequency range indicates that 4ph processes make a substantial contribution to phonon relaxation, making them an important anharmonic scattering mechanism that effectively limits phonon transport. The 3ph and 4ph scattering rates of penta-PdP_2_ are higher than those of penta-PtP_2_ across most of the frequency range, suggesting shorter overall phonon lifetimes and implying the possibility of a lower lattice thermal conductivity for penta-PdP_2_.

To further evaluate the thermal transport properties of penta-XP_2_ (X = Pd, Pt), the temperature-dependent lattice thermal conductivities (*κ*_l_) under 3ph and combined 3ph and 4ph scattering processes are calculated, as shown in Figure 3e. The thermal conductivity obtained under 3ph scattering (*κ*_3ph_) alone exhibits a typical inverse relationship with temperature, characteristic of phonon scattering-dominated behavior. The *κ*_3ph_ of penta-PdP_2_ and penta-PtP_2_ reach 4.41 and 7.87 W/mK at 300 K, respectively, both of which are lower than previously reported results [30]. This reduction results from larger supercells and longer interaction cutoffs, which enhance anharmonic scattering and shorten phonon lifetimes [54]. Across the temperature range, penta-PtP_2_ consistently exhibits higher *κ*_3ph_ than penta-PdP_2_, which aligns with its relatively weaker anharmonicity discussed earlier. Upon inclusion of 4ph scattering, both materials experience a significant reduction in *κ*_3ph+4ph_, as shown in the inset of Figure 3e. The reduction of κ_l_ caused by 4ph scattering increases with temperature, especially above the Debye temperature (307 K for penta-PdP_2_ and 253 K for penta-PtP_2_), highlighting the growing importance of four-phonon processes at elevated temperature. At 300 K, the *κ*_3ph+4ph_ values of penta-PdP_2_ and penta-PtP_2_ reduce to 0.87 and 1.64 W/mK, corresponding to reductions of 80% and 79%, respectively. The strong impact of 4ph scattering, comparable to that observed in other 2D materials such as graphene [55] (~76%), highlights the pronounced anharmonicity in the penta-XP_2_ monolayer. In addition, the κ_l_ under 3ph scattering variation is *T*^−0.972^ for penta-PdP_2_ and *T*^−0.977^ for penta-PtP_2_, and the temperature dependence becomes steeper under combined 3ph and 4ph scattering, with variation as *T*^−1.245^ for penta-PdP_2_ and *T*^−1.256^ for penta-PtP_2_. The stronger suppression originates from the enlarged phase space and increased probability of multi-phonon scattering at higher temperatures, which is consistent with previous reports on MoS_2_ monolayer [56]. To further analyze the underlying scattering mechanisms, the 4ph scattering rates are separated into Normal and Umklapp processes, as well as into combination, redistribution, and splitting processes, as shown in Figure S2. The Umklapp scattering is comparable in magnitude to Normal scattering and acts as the primary source of thermal resistance. The redistribution process fulfills momentum conservation more efficiently and hence is the leading channel in four-phonon scattering. These two types of scattering jointly reduce the lattice thermal conductivity of penta-XP_2_ (X = Pd, Pt). The 4ph scattering rates also lead to a pronounced reduction in the representative phonon mean free path (MFP), as shown in Figure 3f. The inclusion of 4ph processes significantly shifts the cumulative thermal conductivity toward shorter MFPs, indicating a suppression of long-range phonon transport. Such suppression markedly reduces the overall *κ*_l_, which is generally favorable for enhancing the thermoelectric figure of merit (*ZT*) when paired with optimized electronic transport properties.

**Figure 4:**
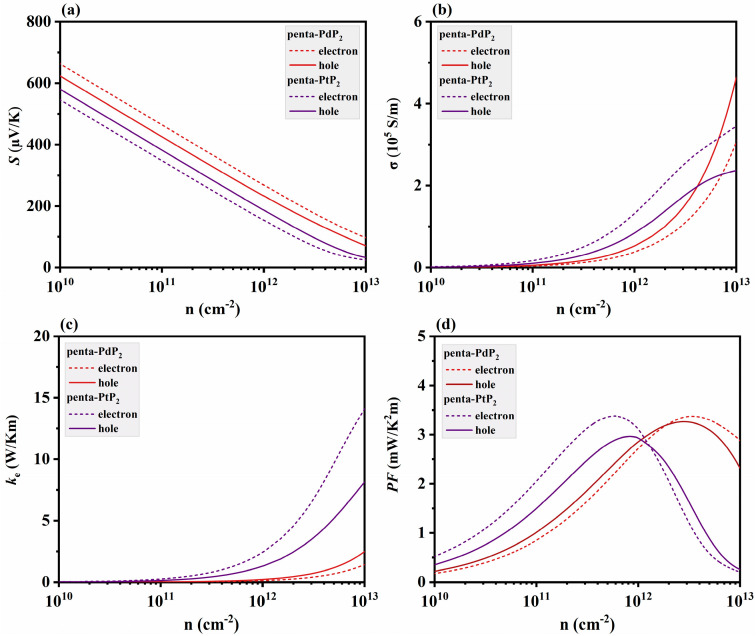
(**a**) Seebeck coefficient (*S*), (**b**) electrical conductivity (*σ*), (**c**) electronic thermal conductivity (*κ*_e_), and (**d**) power factor (*PF*) of penta-XP_2_ (X = Pd, Pt) monolayers as functions of carrier concentration for both electron and hole doping.

Considering the electron–phonon interaction (EPI) effects, the electronic transport properties of penta-XP_2_ (X = Pd, Pt) are shown in Figure 4. As illustrated in Figure 4a,b, the Seebeck coefficients (*S*) of both materials decrease with increasing carrier concentration, and the electrical conductivities (*σ*) exhibit an opposite upward trend, consistent with the typical behavior of thermoelectric semiconductors [57,58]. With EPI included, penta-XP_2_ exhibit higher *S* and lower *σ* than previously reported [30], primarily because phonon scattering reduces carrier mobility, thereby suppressing *σ* and enhance *S*. The electrons in penta-PdP_2_ exhibit higher *S* and lower *σ* than holes, whereas holes in penta-PtP_2_ display higher *S* and lower *σ* than electrons, reflecting the steeper valence band in penta-PdP_2_ and the steeper conduction band in penta-PtP_2_, as shown in Figure 1f,g. A steeper band slope increases the energy dependence of carrier velocity, which enhances *S*, increases the effective mass, and reduces *σ* [59]. Figure 4c shows that electronic thermal conductivity (*κ*_e_) increases significantly with carrier concentration, primarily due to its positive correlation with *σ*, and penta-PtP_2_ generally exhibits higher *κ*_e_ than penta-PdP_2_, with the difference more pronounced under electron doping. The power factor (*PF*) is determined by the combined effects of the *S* and *σ*. As shown in Figure 4d, the *PF* of penta-XP_2_ (X = Pd, Pt) increases with carrier concentration at first and then decreases at 300 K, which is a typical trend for thermoelectric materials. With EPI effects included, the maximum *PF* reaches about 3.38 mW/mK^2^ for electron-doped penta-PdP_2_ and 3.36 mW/mK^2^ for electron-doped penta-PtP_2_, with peak values for electron and hole doping differing only slightly. The *PF* of penta-XP_2_ is within the upper range of reported values for 2D thermoelectric materials, such as monolayer PtSe_2_ [60] and ZnGeSTe [33] (1–4 mW·m^−1^·K^−2^ at room temperature), indicating strong potential for enhanced thermoelectric performance when carrier concentrations are optimized.

**Figure 5:**
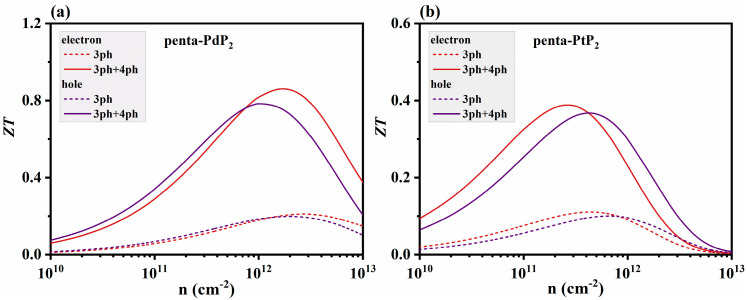
Thermoelectric figure of merit (*ZT*) of (**a**) penta-PdP_2_ and (**b**) penta-PtP_2_ as a function of carrier concentration at 300 K, considering three-phonon (3ph) scattering and combined three-phonon and four-phonon (3ph + 4ph) scattering.

By utilizing the captured thermal and electrical transport parameters, the thermoelectric figure of merit (*ZT*) of penta-XP_2_ (X = Pd, Pt) is illustrated in Figure 5. The thermal conductivity is obtained by adding the *κ*_e_ to the *κ*_l_, with *κ*_l_ evaluated separately for 3ph scattering and combined 3ph and 4ph scattering. The *ZT* values of penta-PdP_2_ and penta-PtP_2_ are approximately 0.21 and 0.11 at room temperature when only 3ph scattering is considered. With the inclusion of 3ph and 4ph scattering, both *ZT* values of penta-XP_2_ increase notably to about 0.86 and 0.34, accompanied by a broader optimal carrier concentration range. This indicates that 4ph anharmonic effects in XP_2_ not only preserve but also enhance thermoelectric performance by optimizing the distribution of phonon scattering pathways, thereby strengthening and stabilizing peak *ZT* regions and providing theoretical support for achieving efficient thermoelectric conversion over a wide carrier concentration range.

## 4. Conclusions

This work comprehensively investigates the thermal transport and thermoelectric properties of penta-XP_2_ (X = Pd, Pt) monolayers using first-principles calculations, incorporating 4ph scattering and EPI effects. Both materials are dynamically and thermally stable, as confirmed by the absence of imaginary phonon frequencies and minimal structural distortion in AIMD simulations. Each penta-XP_2_ monolayer is a direct band gap semiconductor. Phase space and mode-resolved scattering rate analyses establish that 4ph processes contribute on a scale comparable to 3ph scattering, with Umklapp and redistribution processes dominating normal scattering. These scattering channels generate substantial thermal resistance and alter phonon population distributions, leading to further suppression of *κ*_l_. At 300 K, *κ*_l_ decreases from 4.41 to 0.87 W/mK for penta-PdP_2_ and from 7.87 to 1.64 W/mK for penta-PtP_2_, corresponding to reductions of 80% and 79%, respectively. The inclusion of 4ph processes significantly shifts the cumulative thermal conductivity toward shorter MFPs and suppresses long-range phonon transport, offering potential for enhanced thermoelectric performance. The steeper valence band in penta-PdP_2_ leads to higher *S* and lower *σ* for electrons than for holes, and the steeper conduction band in penta-PtP_2_ results in higher *S* and lower *σ* for holes. With the inclusion of 4ph scattering, the figure of merit *ZT* of penta-PdP_2_ and penta-PtP_2_ increases from 0.21 to 0.86 and from 0.11 to 0.34, respectively, along with a broadened optimal carrier concentration range. Our present results demonstrate that 4ph anharmonic effects in penta-XP_2_ not only preserve but can improve thermoelectric performance by optimizing phonon scattering pathways, providing fundamental insights for their potential in efficient nanoscale thermal management and energy conversion applications.

## Data Availability

Data will be made available on request.

## Supplementary Materials

The following supporting information can be downloaded at: https://www.mdpi.com/article/10.3390/nano15181396/s1, Figure S1: Electronic band structures of penta-PdP_2_ (PBE functional: (a), HSE06 functional: (b)) and penta-PtP_2_ (PBE functional: (c), HSE06 functional: (d)) calculated with and without spin–orbit coupling (SOC). The Wannier-interpolated bands are also shown for PBE functional results to verify interpolation accuracy. The inset of (c) highlights the band features near the Fermi level; Figure S2: (a,b) Mode-resolved four-phonon 4ph scattering rates of penta-XP_2_ (X = Pd, Pt) monolayer under Normal and Umklapp processes. (c,d) Mode-resolved four-phonon 4ph scattering rates of penta-XP_2_ (X = Pd, Pt) monolayer with individual contributions from combination (λ + λ′ + λ″ → λ‴), redistribution (λ + λ′ → λ″ + λ‴), and splitting (λ → λ′ + λ″ + λ‴) scattering processes.
