# Supplementary material for: Impact of Four-Phonon Scattering on Thermal Transport and Thermoelectric Performance of Penta-XP2 (X = Pd, Pt) Monolayers
Source: Nanomaterials (Basel). 2025 Sep 11;15(18):1396. doi: 10.3390/nano15181396 (PMC12472265; doi:10.3390/nano15181396)
Supplement: Supplementary file 1 [file nanomaterials-15-01396-s001.zip › nanomaterials-3843453-supplementary.pdf]

Supplemental Material for "Impact of Four-Phonon Scattering on Thermal Transport and Thermoelectric Performance of Penta-XP_2 (X = Pd, Pt) Monolayers"

Yangshun Lan[1], Yueyu Zhang[1], Honggang Zhang[1], Ping Wang[1], Ning Wang[1], Yangjun Yan[1], Xiaoting Zha[1], Changchun Ding[1]; Yuzhi Li[2, *], Chuanfu Li[1, *], Yunjun Gu[3]and Qifeng Chen[4, *]

[1] Key Laboratory of High Performance Scientific Computation, School of Science, Xihua University, Chengdu 610039, China

[2] School of Physics and Electronic Engineering, Sichuan University of Science & Engineering, Yibin 644000, China

[3] National Key Laboratory for Shock Wave and Detonation Physics Research, Institute of Fluid Physics, Chinese Academy of Engineering Physics, Mianyang 621900, China

[4] School of Mathematics and Physics, Southwest University of Science and Technology, Mianyang 621010, China

[*] *Corresponding authors. E-mail: liyzh@suse.edu.cn, lichuanfu_com@126.com, chenqf01@gmail.com*

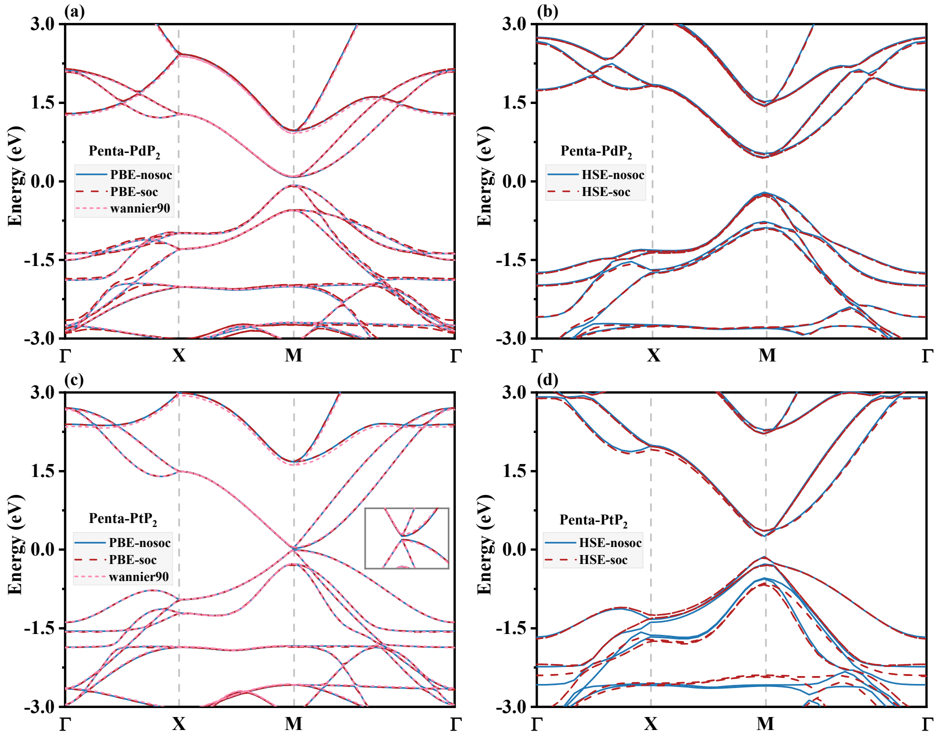

Figure S1. Electronic band structures of penta-PdP_2 (PBE functional: (a), HSE06 functional: (b)) and penta-PtP_2 (PBE functional: (c), HSE06 functional: (d)) calculated with and without spin–orbit coupling (SOC). The Wannier-interpolated bands are also shown for PBE functional results to verify interpolation accuracy. The inset of (c) highlights the band features near the Fermi level.

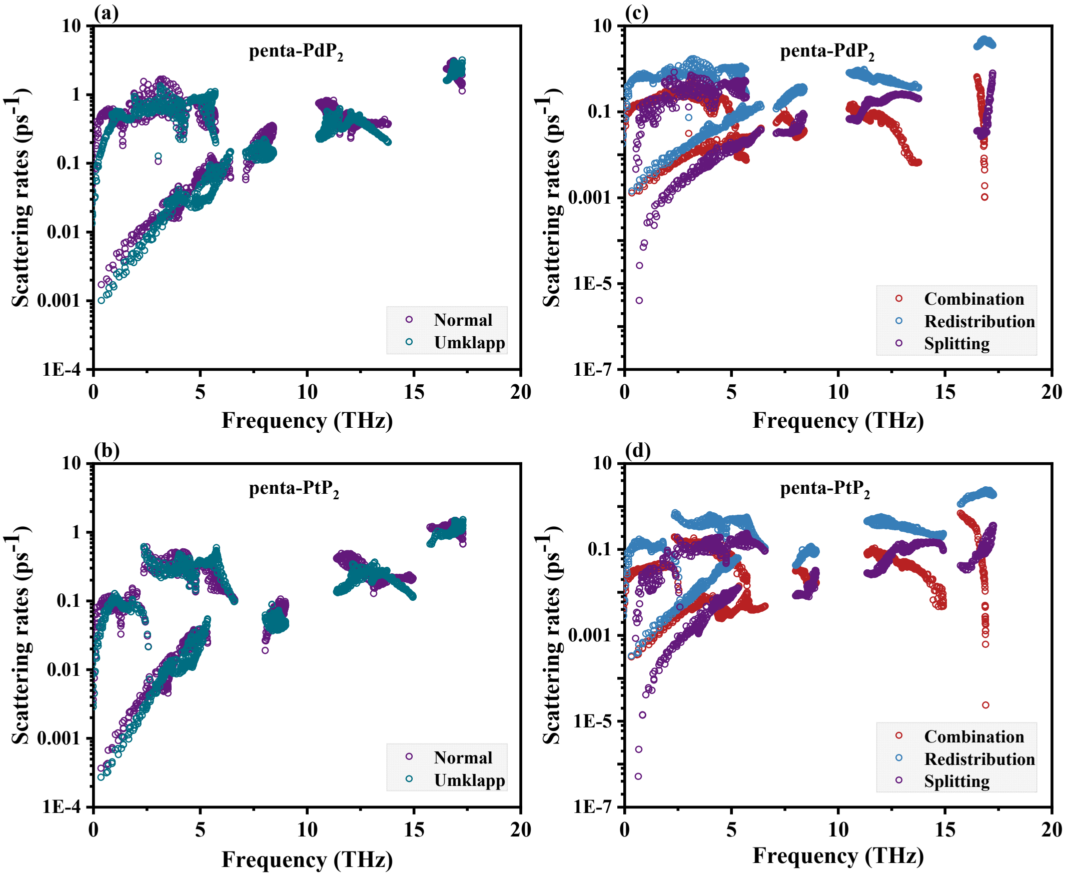

Figure S2. (a, b) Mode-resolved four-phonon 4ph scattering rates of penta-XP_2 (X = Pd, Pt) monolayer under Normal and Umklapp processes. (c, d) Mode-resolved four-phonon 4ph scattering rates of penta-XP_2 (X = Pd, Pt) monolayer with individual contributions from combination ($\lambda + \lambda' + \lambda'' \rightarrow \lambda'''$), redistribution ($\lambda + \lambda' \rightarrow \lambda'' + \lambda'''$), and splitting ($\lambda \rightarrow \lambda' + \lambda'' + \lambda'''$) processes.
